# Utility of a Multi-Marker Panel with Ultrasound for Enhanced Classification of Adnexal Mass

**DOI:** 10.3390/cancers16112048

**Published:** 2024-05-28

**Authors:** Andrew N. Stephens, Simon J. Hobbs, Sung-Woog Kang, Martin K. Oehler, Tom W. Jobling, Richard Allman

**Affiliations:** 1 Cleo Diagnostics Ltd., Melbourne 3000, Australia; simon.hobbs@cleodx.com (S.J.H.); richard.allman@cleodx.com (R.A.); 2 Hudson Institute of Medical Research, Clayton 3168, Australia; kansu353@gmail.com; 3 Department of Molecular and Translational Sciences, Monash University, Clayton 3168, Australia; 4 Department of Gynecological Oncology, Royal Adelaide Hospital, Adelaide 5000, Australia; oehler.mk@gmail.com; 5 Robinson Institute, University of Adelaide, Adelaide 5000, Australia; 6 Department of Gynecological Oncology, Monash Medical Centre, Bentleigh East 3165, Australia; tjobling@bigpond.net.au

**Keywords:** ovarian, cancer, CXCL10, biomarker, triage, malignant, benign, diagnostic

## Abstract

**Simple Summary:**

Pre-surgical evaluation of a sonographically indeterminate adnexal mass is a complex process, and surgical referral relies on subjective evaluation of multiple clinical parameters. The requirement to mitigate malignancy risk means that the vast majority of post-surgical diagnoses are ultimately benign. Conversely, less than half of cancer patients receive a primary referral to an oncology expert, which can adversely affect their long-term outcome. In this study, we highlight the combination of transvaginal ultrasound with a multi-biomarker panel (MMP) to accurately identify and differentiate benign from all stages of malignant disease. The MMP index combined with transvaginal ultrasound (TVU) was superior to TVU plus CA125, particularly for the identification of early-stage malignancies. Incorporation of the MMP index into TVU-driven workflows can provide improved accuracy for the pre-surgical assessment of an adnexal mass, enabling greater confidence for subsequent patient triage.

**Abstract:**

Pre-surgical clinical assessment of an adnexal mass typically relies on transvaginal ultrasound for comprehensive morphological assessment, with further support provided by biomarker measurements and clinical evaluation. Whilst effective for masses that are obviously benign or malignant, a large proportion of masses remain sonographically indeterminate at surgical referral. As a consequence, post-surgical diagnoses of benign disease can outnumber malignancies up to 9-fold, while less than 50% of cancer cases receive a primary referral to a gynecological oncology specialist. We recently described a blood biomarker signature (multi-marker panel—MMP) that differentiated patients with benign from malignant ovarian disease with high accuracy. In this study, we have examined the use of the MMP, both individually and in combination with transvaginal ultrasound, as an alternative tool to CA-125 for enhanced decision making in the pre-surgical referral process.

## 1. Introduction

Current 5-year survival rates for ovarian cancer languish below 50%, making it one of the most lethal gynecological malignancies [1]. Early diagnosis prior to extra-ovarian spread provides the greatest benefit to patients, with above-90% 5-year survival [1]. The initial identification of ovarian cancer presents a significant clinical challenge; however, due to its low incidence, non-specific presentation, and lack of effective diagnostic testing. Whilst population-based screening may ultimately reduce overall mortality, there is currently no effective screening modality to identify early, low-volume malignant disease.

Critical for effective clinical management is the initial assessment and risk stratification for patients with a suspected adnexal mass. The standard work-up includes an evaluation of symptoms, a physical examination, family and medical history, transvaginal ultrasound (TVU), and biomarker measurements. Among these, TVU is recognized as the most effective approach for first-pass investigation [2,3], and subjective evaluation by an expert sonographer (with appropriate training and experience in gynecology) is particularly useful for the differentiation of benign from malignant adnexal masses before surgery. For the identification of ovarian malignancies, ultrasound provides a more effective first-pass evaluation than CA125 [4]. Initial TVU risk assessment is typically based on visible morphological features (e.g., cystic or solid regions, vascularity, presence of papillary projections or peritoneal ascites [5]) and classifies masses as “almost certainly benign”, “suspicious for malignancy” or “indeterminate” [6]. Whilst TVU generally provides an accurate evaluation, the subjective nature of assessment means that interpretation can vary between practitioners [6,7]. As the majority of adnexal masses observed by transvaginal ultrasound (TVU) in postmenopausal women will require surgical intervention [2], it is important to establish an accurate risk of malignancy early and triage patients appropriately.

In the absence of a universally adopted classification system, several approaches have been proposed for the imaging-based classification of adnexal masses. Prominent amongst these are the International Ovarian Tumour Analysis (IOTA) Simple Rules [8,9], which classify lesions visualized by ultrasound into one of five categories according to the presence of benign “B” or malignant “M” features. Recent meta-analysis suggests the IOTA simple rules can identify malignancy with high sensitivity (93% [95%CI, 83–97%]) and good specificity (82% [95%CI 62–93%]) [10]. IOTA simple rules are generally used in pre-menopausal patients [11]. A related imaging-based scoring system is the Ovarian-Adnexal Reporting and Data System (O-RADS) [12], which encompasses all risk categories and malignant schemes. IOTA scoring also forms the basis of the Assessment of Different Neoplasias in the Adnexa (ADNEX) model [13], which incorporates the IOTA simple rules, Cancer Antigen 125 (CA125) serum titer, age, and menopausal status to define the likelihood of malignancy. The O-RADS, IOTA, and ADNEX models all provide similar sensitivity/specificity characteristics for the pre-surgical identification of a benign versus malignant adnexal mass [10,14,15,16]. However, more than 20% of the masses remain indeterminate about following these approaches.

The Risk of Malignancy Index (RMI) [5,17] also combines the ultrasound score, CA125 serum titer and menopausal status to assess the risk of malignancy, and is the only internationally recognized scoring system recommended in medical guidelines [2,3,18]. RMI is most useful in post-menopausal women, where a threshold score of 200 is the cut-off for referral to a gynecological oncology specialist [2]. With specificity at 90%, RMI (200) achieves sensitivity between 64% (pre-menopausal) and 72.3% (postmenopausal) for the detection of cancer [15,16]. Similarly, an RMI score between 25 and 200 is considered “intermediate” risk [3,19] and requires a subjective judgement to be made for the next step in the referral pathway. Whilst rapid and direct referral to a gynecological oncology specialist has the greatest benefit for cancer patients [20,21,22,23,24], RMI does not provide sufficient accuracy for the reliable identification of malignancy—particularly in pre-menopausal women and in those with early-stage, low-volume disease [25,26]. As a consequence, less than 50% of cancer patients receive a primary referral to a gynecologic oncology specialist [6,27]. Patients with benign disease may also benefit from a more conservative treatment approach, particularly one focused on fertility preservation [28,29]. However, the clinical requirement to effectively manage risk means that in the US, around nine benign cases are currently diagnosed for every cancer [23,30,31]. Exploratory surgery for ultimately benign masses carries complication rates between 2 and 15% [32], highlighting the need for accurate diagnosis to minimize potentially harmful interventions and improve the referral process [32].

Biomarker-based testing is an adjunct to TVU for pre-surgical triage, and the potential benefit of multimodal tests is recognized in the American College of Obstetricians and Gynaecologists (ACOG) guidelines [2]. Whilst the Risk of Malignancy Algorithm (ROMA) and OVA1^TM^ tests are suggested as potential alternatives to CA125, neither is recommended for use. We recently identified a multi-marker panel (MMP) that provided high sensitivity and specificity for the differentiation of benign from malignant adnexal masses and that could correctly identify the majority of early-stage and low-CA125 tumours [26,33]. However, its performance against TVU—the primary tool used for referral of patients with a suspicious adnexal mass [2,3]—was not assessed. In this study, we have evaluated the use of the multi-marker panel, both individually and in combination with first look TVU, for its potential to provide improved identification of malignancy for pre-surgical triage.

## 2. Materials and Methods

### 2.1. Patient Cohort

Data were obtained from a recently published retrospective cohort analysis, assembled from multiple centres in Victoria, Australia, between 2007 and 2021 [26]. Relevant details of the cohort are provided in Table 1. A total of 169 samples, for which ultrasound, CA125 titer, RMI2 score, and Multi-Marker Panel (MMP) Index scores were available, were eligible for inclusion in this study. All patients included in the study provided informed written consent (ethical approval numbers: HREC #06032C, #02031B; Southern Health Human Research Ethics Committee). In brief, samples of EDTA-chelated plasma were collected from chemo-naïve patients who underwent surgery in specialist gynecological oncology clinics following referral for suspected malignancy. All patients were anaesthetized at the time of blood collection. Post-surgical diagnoses were confirmed from hospital records, and a review of ultrasound imaging according to the RMI2 schedule [34] was performed and scored by a gynecological oncology specialist. All other details were as previously described [26].

### 2.2. Predictive Scoring and Cut-Off Values

Predictive scores for transvaginal ultrasound imaging (1 or 4), CA125 titer, calculated RMI2, and the recently published MMP index [26] were obtained from Stephens et al. [26]. The cutoff values used for CA125 (post-menopausal > 35 U/mL; pre-menopausal > 200) were based on the most recent American College of Gynecology (ACOG) guidelines [2]. Ultrasound scoring was performed by a gynecological oncology specialist. The risk of Malignancy Index (RMI) was calculated as previously described [35] using the formula:

RMI = ultrasound score × menopausal status × serum CA125

where the ultrasound score is 1 or 4, menopausal status is 1 (pre) or 4 (post), and serum CA125 is in units/mL. The cutoff value for RMI (>200) was used as defined in the published literature [17]. The MMP index was calculated using a multivariate logistic regression model [26], which combines 5 individual biomarker measurements into a single score. A cutoff score of <3.684 that provided 95% sensitivity/specificity for discrimination between samples from patients with benign versus malignant disease was previously determined [26].

### 2.3. Statistical Analyses

All statistical analyses, including logistic regression, Receiver-Operator Curve (ROC) generation, determination of odds ratios (OR), classification tables, predictive power, and *t*-tests, were performed using GraphPad Prism v10.0.3 (275) (Boston, MA, USA). Logistic regression was used to estimate the pooled sensitivity, specificity, positive and negative likelihood ratios (PLRs and NLRs, respectively), diagnostic odds ratio (DOR), and their respective 95% confidence intervals (CIs). Receiver operator characteristic (ROC) curves and the area under the curve (AUC) method were used to estimate diagnostic performance. Sensitivity, specificity, and positive and negative predictive values (PPV and NPV) were calculated as described [36].

## 3. Results

### 3.1. Cohort Features

The characteristics of the cohort data accessed for this study are provided in Table 1 (additional details can be found in [26]). A total of 169 patient samples were recovered with complete biomarker and ultrasound information and were eligible for inclusion in the study. All patients were from a retrospectively collected cohort, previously triaged to a gynecological oncology specialist centre for suspected ovarian malignancy following suspicious ultrasound findings. Within this cohort were a total of 56 ovarian malignancies (33%) and 113 benign (67%) cases, with 39% derived from pre- and 61% from post-menopausal women, respectively (Table 1). Malignancy was more commonly diagnosed in post-menopausal women (~46% of samples) compared to pre-menopausal (~14% of samples). The vast majority of cancer diagnoses in post-menopausal patients were of serous epithelial pathology (87%), whilst pre-menopausal patients tended to have other types (endometroid, clear cell), but nearly all cancers diagnosed were high grade (grades 2–3) in any case. Amongst the samples included were 7 (~4%) stage I ovarian cancers, which were more likely to be diagnosed in pre-menopausal (44%) than post-menopausal (6%) women. The ultrasound score was proportionally higher in post-menopausal patients (50% with ultrasound score = 4) than pre-menopausal patients (24% with ultrasound score = 4). Overall, 47% of the cohort (80 of 169) had known genetic abnormalities at the time of diagnosis, with pre-menopausal patients more likely (~65%) than post-menopausal patients (~36%) to have a known mutation, although over half (52%) of post-menopausal patients had an “unknown” mutational status, compared with only 24% of pre-menopausal patients.

### 3.2. Scoring Distribution between Disease Groupings

**Figure 1:**
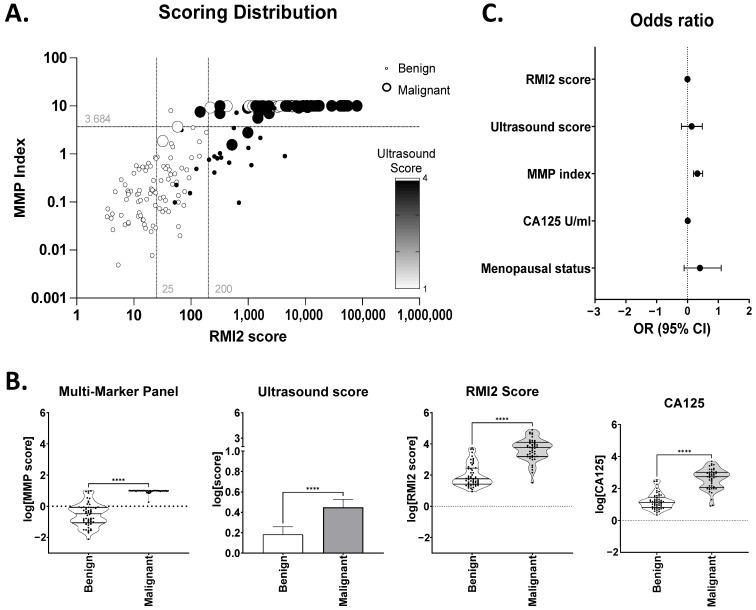
Relationship between Ultrasound, RMI2 score and MMP index. (**A**) MMP index vs. RMI score were plotted for each patient. Each data point was coloured according to ultrasound score of 1 (white) or 4 (black). Circle size indicates benign (small) or malignant (large) status. Grey lines indicate RMI score cut-off values at 25 and 200. (**B**) Comparison of each MMP index, ultrasound score, RMI2, and CA125 between benign and malignant samples. **** *p* ≤ 0.0001. (**C**) Odds ratio +/− 95% confidence intervals for parameters considered in logistic regression modelling.

The relationship between ultrasound score (used for initial triage of patients to the specialist centre and assigned on review by the specialist consultant), calculated RMI2 score, and MMP index [26] for the combined cohort is shown in Figure 1. A largely linear relationship between RMI2 and the MMP index was observed (note: the MMP index has a maximum score of 10), indicating a good correlation between the two. Ultrasound score alone, a major component of initial patient triage, was less useful; an ultrasound score of 4 (indicating malignancy) was associated with ~35% of benign cases, whilst ~25% of malignant cases had ultrasound scores of 1 (Figure 1A). Whilst mean scores for each of the MMP index, RMI2, ultrasound, or CA125, were significantly different between groups (Figure 1B), significant overlap was evident between benign and malignant cases for RMI2, CA125 and ultrasound. Only the MMP index provided a clear separation between the groups.

Odds ratios were calculated to assess the relative contribution of variables and their predictive power for differentiation between benign and malignant groups (Figure 1C). Age at diagnosis, menopausal status, BMI (<30 or >30), and genetic risk factors (BRCA1+. BRCA2+, Lynch Syndrome) were all considered, in addition to CA125 and the previously calculated scores for ultrasound, RMI2, and MMP index. Amongst the parameters included in the model, only the MMP index (OR 2.09; 95%CI 1.57–3.09; *p* < 0.0001) was significant. Menopausal status (OR 2.53; 95%CI 0.78–12.48; *p* = 0.1) was not significant in this small dataset but trended towards a positive impact on the differentiation of benign from malignant disease (Figure 1C). Neither ultrasound score (OR 1.37; 95%CI 0.67–3.04; *p* = 0.42), RMI2 score (OR 1.0; 95%CI 0.99–1.00; *p* = 0.13) nor CA125 (OR 1.02; 95%CI 1.00–1.05; *p* = 0.09) contributed significantly to differentiation between groups (Figure 1C), reflecting the “high risk” nature of this cohort already triaged to a gynecological oncology clinic based on elevated RMI and CA125 scores [26]. No significant discrimination was evident for age (OR 0.93; 95%CI 0.83–1.03), genetic status (OR 0.06; 95%CI 0.06–6.87), or BMI (OR 0.18; 95%CI 0.01–1.16) between groups although BMI > 30 showed a trend towards a negative impact on group differentiation, suggesting a BMI < 30 may be more commonly associated with benign status. 

**Figure 2:**
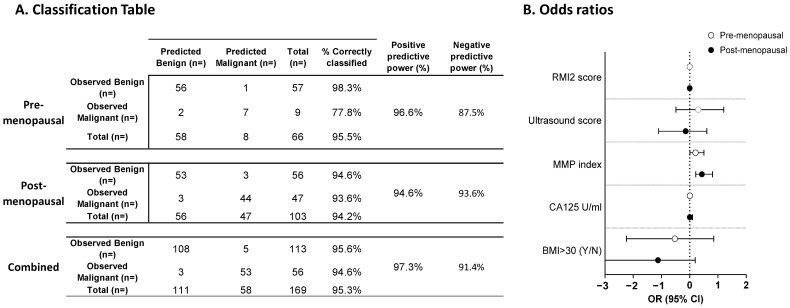
Results of logistic regression analysis. (**A**) Classification table according to logistic regression. Analyses were performed on all samples, or on samples separated by menopausal status. (**B**) Odds ratios +/− 95% confidence intervals associated with pre-menopausal (white circle) or post-menopausal (black circle) status.

As menopausal status is related to disease risk, regression analyses and odds ratios were re-calculated for each of the pre- and post-menopausal groups separately (Figure 2). Age and genetic status were omitted from the model, as they had no clear influence on the outcome. Between 94 and 97% of patients could be correctly classified regardless of menopausal status and achieved similar negative predictive powers (Figure 2A). Positive predictive power was slightly lower in pre-menopausal patients (87.5%) than post-menopausal (93.6%) patients, although this may reflect low numbers in the pre-menopausal cohort; interestingly, ~44% of cancers in the pre-menopausal cohort were stage 1, suggesting reasonable performance for early-stage cancer detection in this group (Figure 2A). The calculation of odds ratios (Figure 2B) again highlighted the MMP index as an important variable for classification (pre-menopausal OR 1.61 95%CI 1.01–3.20, *p* = 0.047); post-menopausal OR 2.71 95%CI 1.63–6.93, *p* = 0.003). Again, no other variables contributed significantly to the classification. Overall, ~95% of samples could be correctly classified with a negative predictive power of 97.3% and a positive predictive power of 91.4% (Figure 2A).

### 3.3. MMP Index Out-Performs RMI2 Score for Differentiation of Benign from Malignant Disease

**Figure 3:**
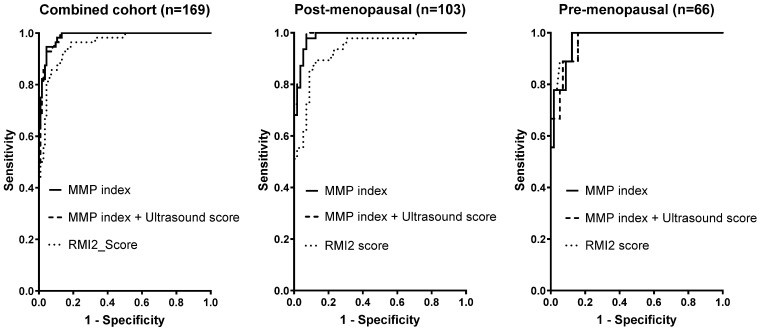
Relative performance for each of the MMP index, RMI score, MMP index, and TVU. ROC curves were individually calculated for each classifier. Cohort details (either total, pre-menopausal, or post-menopausal) are indicated.

The diagnostic performances of the MMP index, RMI2 score, and ultrasound for discrimination between benign and malignant samples were compared using ROC analysis (Figure 3). As ultrasound imaging is currently the clinical gold standard for patient triage, the MMP index was also assessed in combination with ultrasound. Data were evaluated using both the combined cohort and samples separated by pre- or post-menopausal status. Scoring thresholds for ultrasound (1 or 4), RMI2 (>200), and the MMP index (<3.684) were used as previously [17,26]. Metrics for comparison included the area under the curve (AUC), sensitivity/specificity, and negative/positive predictive values (Table 2).

Receiver operator characteristic (ROC) curves were generated for each of RMI, MMP index, and the combination of MMP index and ultrasound score (Figure 3; note that ultrasound alone was omitted from ROC curves for clarity). In the combined cohort, the MMP index, either alone or in combination with ultrasound, achieved a clear increase in overall efficacy compared to the RMI2 score (Figure 3). When separated by menopausal status, the improvement of MMP index over RMI2 was more pronounced in post-menopausal patients; in the pre-menopausal cohort, there was no clear difference between MMP index, RMI2, or the combination of MMP index with ultrasound (Figure 3). The AUC, sensitivity/specificity, positive and negative predictive values (PPV/NPV) for each score and in each cohort subset are presented in Table 2. Overall AUCs were between 0.94 and 0.99 (Table 2), with the exception of ultrasound alone (0.72–0.87). When pre-menopausal patients were considered separately, ultrasound alone provided the greatest specificity and positive predictive value, although it only achieved a sensitivity of 50% (Table 2). There was no obvious advantage identified between the scoring systems for the pre-menopausal cohort.

In post-menopausal patients, the MMP index (AUC 0.99) had a clear advantage over the use of RMI2 (AUC 0.94). In particular, sensitivity/specificity were substantially higher using the MMP index (92.0%/98.1%) than RMI2 (89.9%/77.6%). Whilst the NPV was similar across both measurements, the PPV obtained by the MMP index was also substantially higher than the PPV for RMI2 (97.9% vs. 68.1%, respectively). The combination of MMP index and ultrasound score resulted in a minor improvement in sensitivity, specificity, and positive predictive value, which was evident in the combined cohort only. Overall, however, MMP index alone or in combination with ultrasound achieved the highest level of sensitivity, specificity, PPV, and NPV in this cohort for the discrimination of benign from malignant disease and exceeded the performance of the clinical gold standard RMI score.

### 3.4. Classification Performance for Early-Stage Cancers

**Figure 4:**
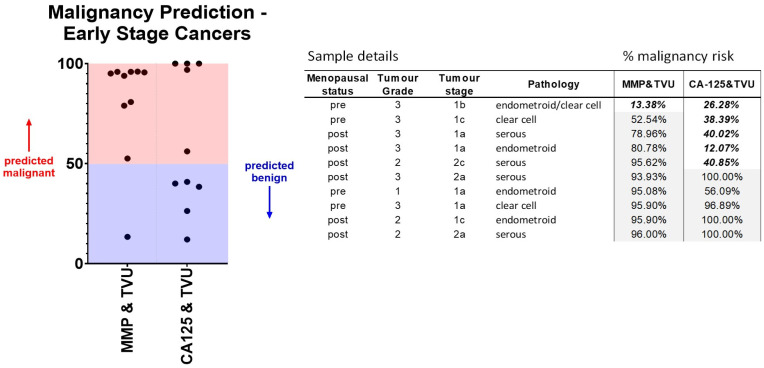
A combination of MMP index with TVU improves classification of stage I–II ovarian malignancy compared to CA125 with TVU. Predicted probabilities for all “early” stage (stages I or II) cancers within the cohort were obtained following logistic regression. Pathology details and the calculated malignancy risk for each sample are shown. A threshold value > 50% suggests malignancy.

Of particular importance in the early triage process is the accurate identification of early-stage or low-volume cancers. Within the cohort studied, 10 patients were diagnosed with either stage I (n = 7) or stage II (n = 3) epithelial ovarian cancers. The predicted probabilities calculated by logistic regression associated with each sample using RMI, MMP index, or MMP index plus TVU are provided in Table 2. Whilst 50% of early-stage samples were predicted as unlikely to be malignant (score < 0.5) using RMI2, almost all were correctly predicted using the MMP index. The addition of ultrasound scores to the MMP index further increased the prediction scores to encompass 90% of early-stage samples (Figure 4).

## 4. Discussion

The management of an adnexal mass at first clinical presentation is a complex and multi-faceted process. Whilst a combination of radiological imaging, biomarkers, patient history, and symptoms underlie the ultimate judgement on whether surgical referral is warranted [6], it is transvaginal ultrasound that provides the most effective tool for the early characterization of an adnexal mass [2,3]. The primary goal of early evaluations is to exclude malignancy, and regardless of the interpretation system used, all patients are categorized into groups with a “low”, “intermediate”, or “high” risk of malignancy and triaged accordingly [6]. The majority of adnexal masses in postmenopausal women will require surgical intervention [2], but following pre-surgical triage, up to 90% of patients are subsequently diagnosed with benign disease [9,37]. In the US alone, there are ~428,000 admissions and ~231,000 surgeries annually for adnexal masses [6,37,38]; yet only ~22,000 malignancies are identified. For the remaining patients, exploratory surgery for benign disease carries between 2 and 15% risk of complications [39,40]. It is therefore critical not only to improve the early identification and referral of patients with a malignant mass but also to eliminate those non-malignant cases where patients may benefit from a more conservative intervention.

Our data demonstrate that the calculation of the MMP index provides substantially improved differentiation of benign from malignant disease compared to the “gold standard” combination of TVU with CA125. Whilst RMI score and MMP index both achieved similar sensitivity and NPVs within this cohort, the specificity and PPV of the MMP index (95.6% and 91.1%) were substantially better than the RMI score (85.2% and 66.1%). This is particularly promising as this study evaluated “high-risk” individuals—already triaged to a gynecological oncology centre based on elevated CA125 and suspicious TVU findings—representing a biased patient group skewed towards high RMI scores. 

The combination of MMP index with TVU also achieved an incremental improvement over MMP index alone, highlighting its potential as an alternative to CA125 for improved pre-surgical triage of adnexal masses. Adjunct biomarker testing for CA125 is recommended in all clinical guidelines for adnexal mass assessment [2,3,18] and is a critical component of both the ADNEX [13] and RMI [5,17] scoring models. Interestingly, however, ultrasound classifications are not significantly impacted by CA125 [41]. Recent analyses suggest RMI should be replaced for pre-surgical triage [16,42], a proposal consistent with indications in the current ACOG guidelines that multi-modal biomarker panels could provide an alternative to CA125 [2]. Our data strongly suggest that the MMP index has high potential to outperform RMI in a non-selected patient cohort, particularly in combination with TVU, as an alternative biomarker test to CA125.

An observation of particular importance was a significant improvement in the identification of early-stage (FIGO stages I–II) ovarian cancers using the MMP index. Compared to the RMI score, which identified 50% of early-stage cancers, the combination of the MMP index with TVU (instead of CA125) in this cohort successfully identified 90% of early cancer cases. Whilst several meta-analyses have confirmed the good overall performance of RMI, ROMA, and ADNEX [16,42], they typically perform poorly for the identification of stage I disease [43,44,45]. Early-stage ovarian cancers rarely display elevated CA125 [46,47], and our previous analyses highlighted that the MMP index could efficiently identify and classify low CA125 malignancies [33]. Taken together, our data demonstrate that the MMP index can provide an effective triage test to correctly identify ovarian cancers at all stages and differentiate them from benign disease.

TVU operator expertise is critical in the pre-surgical determination of malignancy risk [6]. The availability of such expertise varies widely, however [6,12], and is a limiting factor in the overall predictive capacity afforded by TVU. Misclassification of adnexal masses due to interpretative error can be as high as 75% [48]; whilst in post-menopausal women recruited to the UKTOCS study, TVU-based identification of normal ovaries had a specificity of only 47.5% [49]. Standardized scoring systems, including the IOTA simple rules [8,9] and O-RADS-US [12], proposed to maintain consistency in TVU interpretation, typically provide ~80% specificity [4,10,50,51]. Nevertheless, a substantial fraction of adnexal lesions cannot be definitively stratified following TVU [28,52,53]. To improve the characterization of sonographically indeterminate lesions, magnetic resonance imaging (MRI) as part of the O-RADS MRI scoring scheme has been suggested [48]. MRI improves the characterization of adnexal masses and is useful in the diagnosis of malignancy when a sonographically indeterminate mass is observed by TVU [10,54]. Most benign masses will require extended follow-up, however, so the broader application of O-RADS MRI may be minimized due to the morbidity associated with repeated exposure to contrast dye as well as economic considerations [54]. Biomarker testing may provide a more cost-effective option, without a similar risk of morbidity.

This study also confirmed our previous reports [26,33] highlighting the strong negative predictive value of the MMP index to exclude benign cases. Pre-surgical identification of benign masses is essential to exclude malignancy and develop conservative management strategies to minimize morbidity associated with surgical interventions [28,29,32], particularly since most benign cysts do not progress to malignancy [55,56]. Whilst repeat TVU screening for benign disease is usually indicated, the nature and extent of follow-up are not well defined. Current ACOG guidelines recommend follow-up for cysts up to 10 cm in size [2], whilst O-RADS guidelines suggest cysts of less than 3 cm do not require repeat screening [12]. Similarly, the Society of Radiologists in Ultrasound (SRU) only recommends follow-up for cysts over 7 cm in pre-menopausal women, provided “good ultrasound characterization is available” [32]. Recent studies have also suggested extended follow-up for benign adnexal cysts until resolution, potentially up to 8 years after initial identification [57]. An accurate, biomarker-based panel for the early identification of benign disease will not only minimize the large number of false positive cases that proceed to surgery [23,30,31]; but may also provide a far more cost-effective means for ongoing monitoring of non-malignant disease.

## 5. Conclusions

Our data demonstrate superior performance of the MMP index over the current clinical workflow using TVU and CA125 and highlight the combination of the MMP index with TVU to substantially improve the identification of malignancy at an early stage. Incorporation of the MMP index into TVU-driven workflows is likely to improve the clinical management of adnexal masses, particularly for the rapid triage of early-stage cancers as well as the exclusion of non-malignant cases. Prospective cohort studies are now underway to further establish the suitability of the MMP index as a pre-surgical triage panel.

## 6. Patents

Aspects of this study are covered by granted patent 2020404453 and provisional patent 540674PRV.

## Figures and Tables

**Table 1:** Cohort characteristics of patient samples included in this study. All patients were triaged to a gynecological oncology specialist centre for secondary evaluation and surgery.

		All	Pre-Menopausal	Post-Menopausal
# Participants (Total)		n = 169	n = 66	n = 103
Age at diagnosis (years)	median	54	44.6	62.6
	IQ range	47–65	40–49	56–69
Pathology (n=)	benign	113	57	56
	malignant	56	9	47
Tumour type (n=)	Serous	44	4	40
	endometroid	4	1	3
	clear cell	3	2	1
	mixed epithelial	5	2	3
Grade (n=)	1	2	1	1
	2	5	1	4
	3	49	7	42
Stage (n=)	I	7	4	3
	II	3	nil	3
	III–IV	46	5	41
Genetic Predisposition (n=)	BRCA1	29	16	13
	BRCA2	33	17	16
	other (lynch, BRIP1+, PALB+, VUS)	18	10	8
	wild type	19	7	12
	unknown	70	16	54
Ultrasound score (n=)	1	101	50	51
	4	68	16	52

**Table 2:** Diagnostic parameters for each classifier model according to cohort. AUC, sensitivity, specificity, and PPV/NPV were calculated for each predictor using published cut-off values.

Predictor	Published Cut-Off	Menopausal Status	n=	AUC (95%CI)	Sensitivity %	Specificity %	PPV %	NPV %
Ultrasound		combined	169	0.77 (0.70–0.85)	63.2%	87.1%	76.8%	77.9%
	1 or 4	pre	66	0.87 (0.74–10.0)	50.0%	98.0%	88.9%	86.0%
		post	103	0.72 (0.62–0.82)	67.3%	76.5%	74.5%	69.6%
RMI2 score		combined	169	0.95 (0.93–0.98)	90.2%	85.2%	66.1%	96.5%
	≥200	pre	66	0.98 (0.94–1.00)	85.7%	94.9%	66.7%	98.2%
		post	103	0.94 (0.89–0.98)	88.9%	77.6%	68.1%	92.9%
		combined	169	0.99 (0.98–1.00)	91.1%	95.6%	91.1%	95.6%
MMP index	≥3.648	pre	66	0.97 (0.94–1.00)	85.7%	94.9%	66.7%	98.2%
		post	103	0.99 (0.97–1.00)	92.0%	98.1%	97.9%	92.9%
MMP Index + Ultrasound		combined	169	0.98 (0.97–1.00)	91.2%	96.4%	92.9%	95.6%
	n/a	pre	66	0.97 (0.93–1.00)	85.7%	94.9%	66.7%	98.2%
		post	103	0.99 (0.97–1.00)	92.0%	98.1%	97.9%	92.9%

## Data Availability

The datasets used and/or analysed during the current study are available from the corresponding author upon reasonable request.
